# Foliar Nano-Selenium Modulates Metabolic and Antioxidant Responses in Alfalfa (*Medicago sativa* L.): Integration of Pot and Field Evidence

**DOI:** 10.3390/ijms26189013

**Published:** 2025-09-16

**Authors:** Haiyan Cheng, Huan Yu, Qinyong Dong, Chunran Zhou, Tingjie Huang, Xun Fang, Canping Pan

**Affiliations:** Innovation Center of Pesticide Research, Department of Applied Chemistry, College of Science, China Agricultural University, Yuanmingyuan West Road 2, Beijing 100193, China; haiyancheng@cau.edu.cn (H.C.); yhteawork@163.com (H.Y.); dontinue@126.com (Q.D.); chunranzhou@cau.edu.cn (C.Z.); takeyourtimehtj@163.com (T.H.); fangxun@cau.edu.cn (X.F.)

**Keywords:** nano selenium, alfalfa, growth performance, nutritional components

## Abstract

Alfalfa (*Medicago sativa* L.), as a globally crucial high-quality forage crop, frequently suffers from yield reduction and quality deterioration due to environmental stressors such as drought and salt. Nano-selenium (NSe) offers a viable solution to mitigate this challenge. However, the multi-level regulatory mechanisms of NSe in alfalfa remain unclear. Foliar NSe modulates nitrogen metabolism, antioxidant defense, and rhizosphere microbial community collaboration to enhance alfalfa yield and quality. Pot experiments demonstrated that foliar NSe (1–20 mg/L) enhanced seedling growth, elevated nutrient biosynthesis (soluble protein, amino acids), and boosted antioxidant capacity via activation of superoxide dismutase and glutathione peroxidase. Metabolomics in field trials revealed shoot-root metabolic partitioning: shoots were upregulated in α-linolenic acid metabolism (jasmonic acid, methyl jasmonate), while roots enriched amino acid biosynthesis (proline, arginine), achieving a synergistic enhancement between aboveground and belowground processes. Microbial community analysis indicated Actinobacteria enrichment and elevated soil urease activity in NSe-treated groups. These findings demonstrate that NSe coordinates carbon-nitrogen metabolism with antioxidant pathway activation to synergistically enhance alfalfa growth performance and nutritional quality.

## 1. Introduction

Alfalfa (*Medicago sativa* L.) is a widely cultivated high-quality legume forage that serves as an excellent feed source for ruminants due to its rich nutritional content (proteins, amino acids, and vitamins). Alfalfa is renowned as the “Queen of Forages” for its superior quality, high yield, and environmental adaptability. Alfalfa has become pivotal for sustainable livestock production. Alfalfa partners with rhizobia bacteria to fix nitrogen from the air. Its strong root system helps this natural fertilizer-making process work better. Together, these traits make alfalfa a key crop for farmers to enhance soil health and maintain productive fields [1]. Alfalfa quality is regulated by critical components, including fiber (acid detergent fiber, ADF; neutral detergent fiber, NDF), crude protein (CP), starch, and Ash. ADF and NDF contain cellulose, hemicellulose, and lignin. However, abiotic stresses, particularly pesticide stress [2], along with drought [3] and heavy metal toxicity [4], substantially impair the yield and nutritional quality of alfalfa, consequently destabilizing the forage supply chain. There is a pressing need to develop scientifically validated strategies for enhancing alfalfa nutritional quality and growth performance.

Selenium (Se) is a critical element for plant and animal growth and stress resistance. Se facilitates heavy metal detoxification and metabolic regulation in plants through its antioxidant properties [5]. Extensive studies have demonstrated the efficacy of Se in enhancing crop quality (e.g., soybean [6], cucumber [7]). It achieves this primarily by improving nitrogen assimilation efficiency, thereby elevating amino acid and protein contents. Concurrently, Se modulates plant signal transduction pathways to regulate the synthesis and accumulation of secondary metabolites. Crucially, Se-driven quality enhancement is inherently linked to stress resistance augmentation. As a structural component of glutathione peroxidase (GSH-Px), Se enhances antioxidant capacity and osmotic adjustment, effectively counteracting the adverse impacts of biotic (e.g., pathogens) and abiotic stresses (e.g., drought [8], salinity [9], heavy metal toxicity [10]). Nano-selenium (NSe) has emerged as an ideal candidate for crop health management due to its high bioavailability, low toxicity, and enhanced bioactivity [11]. Li et al. [12] reported that NSe stimulates the glutamine synthetase/glutamate synthase (GS/GOGAT) cycle in tea plants to boost the synthesis and accumulation of amino acids (e.g., glutamate, arginine), while improving the ascorbate-glutathione (ASA-GSH) cycle to scavenge reactive oxygen species (ROS), thereby mitigating pesticide-induced oxidative damage. Zhou et al. [13] demonstrated that foliar NSe application accelerated wheat development by stimulating amino acid and phenylpropanoid biosynthetic pathways. NSe also enhances rhizosphere soil enzyme activities and beneficial microbial abundance to mitigate bensulfuron-methyl phytotoxicity. Foliar selenium significantly improves field crop yield and nutritional quality: Xia et al. [14] reported that foliar application in wheat was significantly more effective than soil application in boosting grain Se accumulation, amino acids, and soluble protein content. Similarly, Pasković et al. [15] confirmed that foliar Se in olive trees increased amino acids (tryptophan, tyrosine) and elevated antioxidants such as oleuropein and flavonoids. Existing studies have demonstrated that selenium can promote the growth of alfalfa. For instance, Hu et al. [16] found that sodium selenite (Na_2_SeO_3_) enhances its stress resistance by boosting carbohydrate metabolism.

Building on the biofortification potential of NSe, this study integrated pot and field trials with multi-omics analyses (physiological-biochemical, metabolomic, and microbiome). These approaches systematically elucidated foliar NSe-mediated growth and metabolic regulation in alfalfa. We aimed to: (1) Determine optimal NSe concentrations that enhance growth performance and nutritive value; (2) Examine NSe-driven regulation of amino acid biosynthesis and antioxidant-hormonal networks (jasmonic acid, JA/salicylic acid, SA); (3) Assess its potential modulation of rhizosphere microenvironments (soil properties and microbial communities).

## 2. Results

### 2.1. Effect on Alfalfa Growth

**Figure 1:**
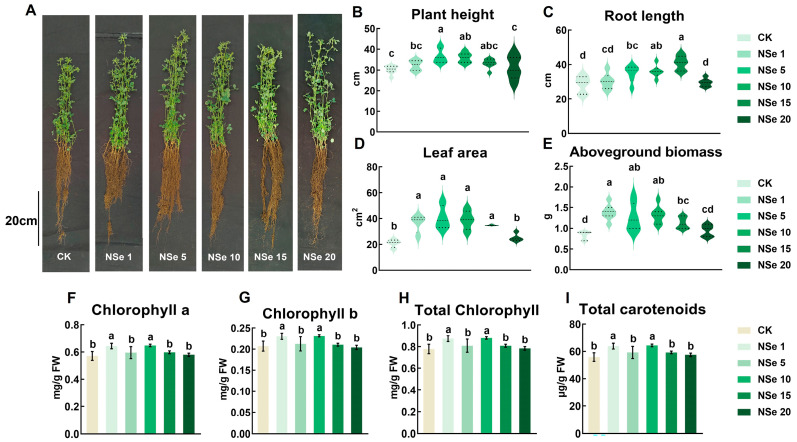
Effects of different treatments on alfalfa growth and photosynthetic pigments (**A**) Morphology, (**B**) Plant height, (**C**) Root length, (**D**) Shoot fresh weight, (**E**) Leaf area, (**F**) Chlorophyll a content, (**G**) Chlorophyll b content, (**H**) Total chlorophyll content, (**I**) Total carotenoid content. Data are presented as mean ± SEM. In panels (**B**–**E**), the dashed lines (from top to bottom) indicate the upper quartile (75th percentile), median (50th percentile), and lower quartile (25th percentile). Different letters indicate significant differences at *p* < 0.05.

The effects of NSe on seedling growth were evaluated by measuring plant height, root length, and leaf area in alfalfa (Figure 1). Phenotypic analysis revealed enhanced root system vigor in NSe-treated plants (Figure 1A). The biomass of NSe-treated alfalfa was significantly higher than that of the control, exhibiting an initial increase followed by a decline as NSe concentrations rose. At 1–15 mg/L NSe, plant height (7.2–22%), root length (6.3–44%), leaf area (67–94%), and aboveground biomass (36–67%) were significantly enhanced compared to the control (Figure 1B–E). However, 20 mg/L NSe showed no significant growth promotion. Photosynthetic pigments (chlorophyll and carotenoids) increased by 11–15% under NSe (1 and 10 mg/L) compared to the control.

### 2.2. Effects on Alfalfa Quality Components

**Figure 2:**
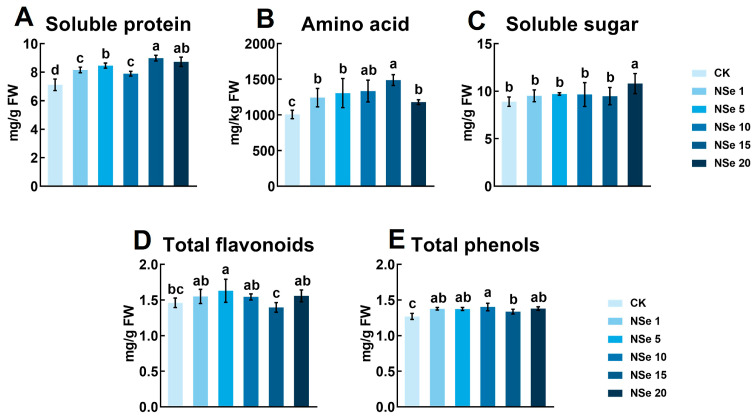
Effects of different treatments on alfalfa quality components (**A**) Soluble protein, (**B**) Amino acid, (**C**) Soluble sugar, (**D**) Total Flavonoids, (**E**) Total phenols. Data are presented as mean ± SEM. Different letters indicate significant differences at *p* < 0.05.

Soluble protein, amino acids, soluble sugars, flavonoids, and total phenolics were measured to assess the impact of NSe on physiological metabolism and quality (Figure 2A–E). Foliar NSe (1–20 mg/L) significantly elevated soluble protein (11–26%) and amino acid contents (17–48%) in alfalfa compared to the control. The 15 mg/L treatment showed the highest efficacy (26% for protein, 48% for amino acids). Total phenolics significantly increased across all concentrations (5.3–10%), while flavonoids were significantly enhanced only at 5 mg/L NSe (12%).

### 2.3. Effects on Alfalfa Free Amino Acids

**Figure 3:**
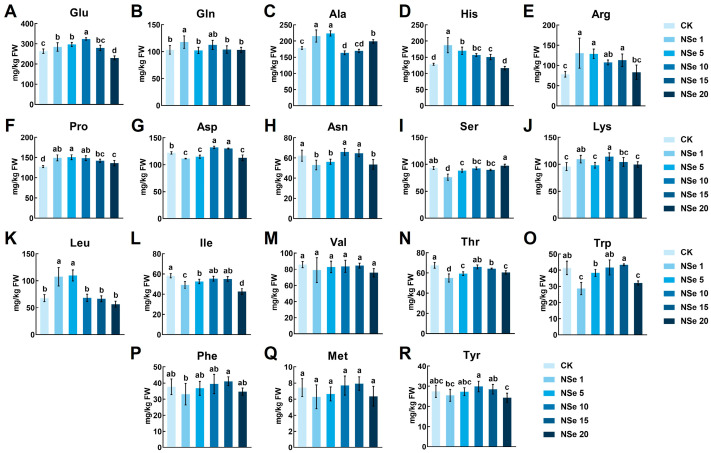
Free amino acid profiles in alfalfa under different treatments (**A**) Glutamate (Glu), (**B**) Glutamine (Gln), (**C**) Alanine (Ala), (**D**) Histidine (His), (**E**) Arginine (Arg), (**F**) Proline (Pro), (**G**) Aspartate (Asp), (**H**) Asparagine (Asn), (**I**) Serine (Ser), (**J**) Lysine (Lys), (**K**) Leucine (Leu), (**L**) Isoleucine (Ile), (**M**) Valine (Val), (**N**) Threonine (Thr), (**O**) Tryptophan (Trp), (**P**) Phenylalanine (Phe), (**Q**) Methionine (Met), (**R**) Tyrosine (Tyr). Data are presented as mean ± SEM. Different letters indicate significant differences at *p* < 0.05.

Eighteen free amino acids (excluding Cys and Gly due to low concentrations) were quantified in alfalfa shoots (Figure 3). Glu, Gln, Ala, His, Arg, Pro, Asp, Lys, and Leu exhibited an initial increase followed by a decline with rising NSe concentration, whereas Ile, Thr, and Trp decreased. Val, Met, and Tyr showed no significant changes. At NSe 1 mg/L, eight amino acids (e.g., Glu, Gln) significantly increased by 7.8–67%, while five (e.g., Asp, Asn) significantly decreased by 8.9–31%, compared to the control.

### 2.4. Effect on Phytohormones

**Figure 4:**
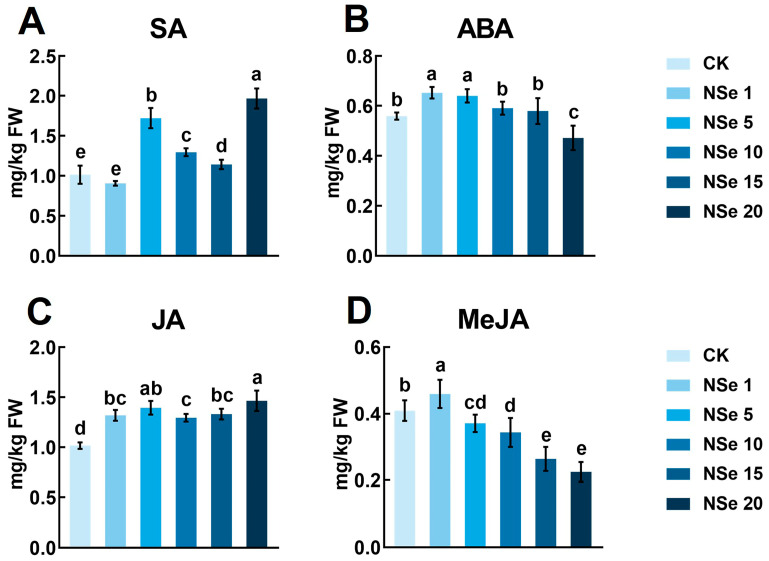
Phytohormone levels in alfalfa under different treatments (**A**) Salicylic acid (SA), (**B**) Abscisic acid (ABA), (**C**) Jasmonic acid (JA), (**D**) Methyl jasmonate (MeJA). Data are presented as mean ± SEM. Different letters indicate significant differences at *p* < 0.05.

Phytohormone levels in shoots are shown in Figure 4A–D. NSe at 5–20 mg/L significantly increased SA by 13–94% compared to the control. Abscisic acid (ABA) significantly increased (1–5 mg/L NSe) but significantly decreased at 20 mg/L. JA significantly increased progressively (28–44%) with rising NSe concentration, while methyl jasmonate (MeJA) decreased (except NSe 1 mg/L).

### 2.5. Effect on Antioxidant System

**Figure 5:**
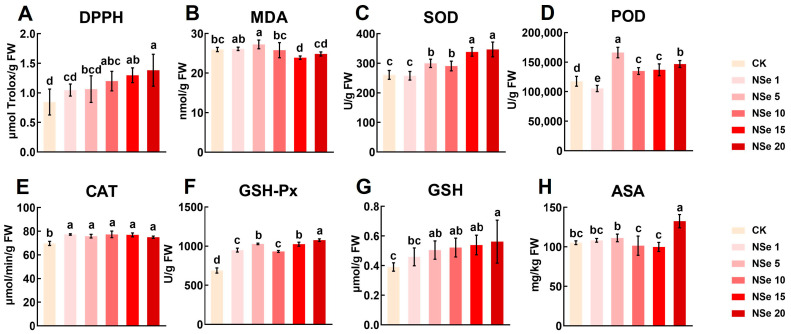
Antioxidant system responses in alfalfa under different treatments (**A**) DPPH total antioxidant capacity, (**B**) Malondialdehyde (MDA) content, (**C**) Superoxide dismutase (SOD) activity, (**D**) Peroxidase (POD) activity, (**E**) Catalase (CAT) activity, (**F**) Glutathione peroxidase (GSH-Px) activity, (**G**) Glutathione (GSH) content, (**H**) Ascorbic acid (ASA) content. Data are presented as mean ± SEM. Different letters indicate significant differences at *p* < 0.05.

To evaluate the effects of NSe treatment on the antioxidant system of alfalfa, we determined the activities of antioxidant enzymes and the levels of antioxidant substances, as shown in Figure 5. Overall, NSe treatment enhanced the antioxidant capacity of alfalfa. DPPH increased with rising NSe concentrations (Figure 5A), with 10–20 mg/L NSe significantly enhancing DPPH activity by 42–64% compared to the control; however, no significant differences were observed among these concentrations. Treatment with NSe significantly increased the activities of key antioxidant enzymes SOD, POD, CAT, and GSH-Px (Figure 5C–F). Concurrently, it elevated the levels of non-enzymatic antioxidants, including GSH and ASA (Figure 5G,H), demonstrating a consistent upward trend across these parameters. The 20 mg/L NSe treatment exhibited optimal efficacy in enhancing antioxidant capacity. Specifically, compared to the control group, NSe (20 mg/L) significantly elevated SOD, POD, CAT, and GSH-Px activities by 33%, 25%, 8%, and 56%, respectively, while significantly increasing GSH and ASA contents by 44% and 26% and concurrently significantly reducing MDA content by 4%.

### 2.6. Effect on Field Trial: Yield and Quality

**Figure 6:**
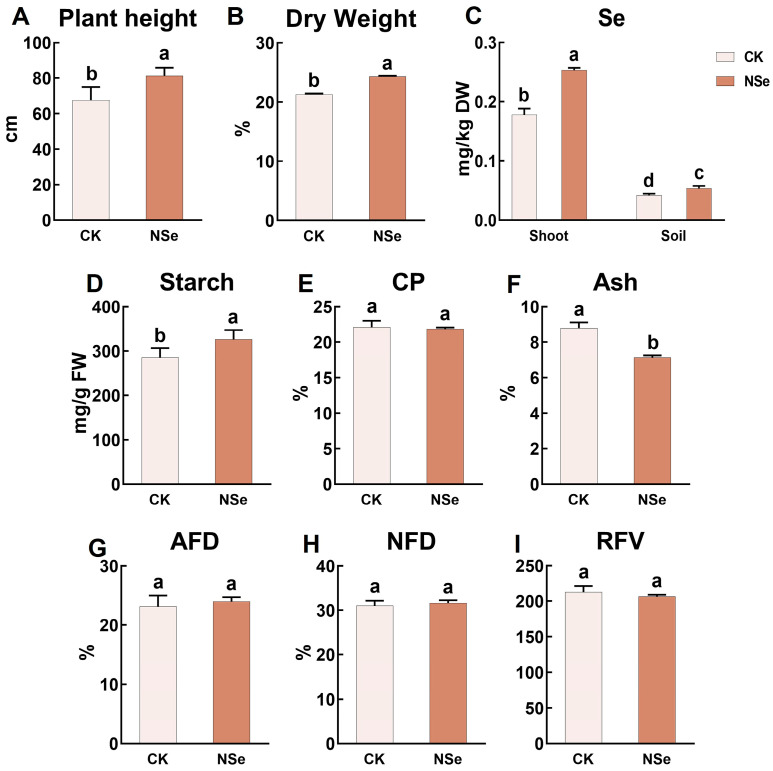
Field trial: Yield and quality parameters of alfalfa (**A**) Plant height, (**B**) Dry weight, (**C**) Se content, (**D**) Starch, (**E**) Crude protein (CP), (**F**) Crude ash, (**G**) Acid detergent fiber (ADF), (**H**) Neutral detergent fiber (NDF), (**I**) Relative feed value (RFV). Data are presented as mean ± SEM. Different letters indicate significant differences at *p* < 0.05.

Field trials were conducted to validate the findings. After NSe application (Figure 6A,B), the plant height of alfalfa significantly increased by 20%, and the dry weight significantly improved by 14% compared to the control. The Se content in aboveground tissues significantly rose by 43% (reaching 0.18 mg/kg dry weight), while the rhizosphere soil Se significantly increased by 27% (0.053 mg/kg dry soil) (Figure 6C). Regarding quality components (Figure 6E,G–I), no significant effects were observed on CP, ADF, NDF, or relative feeding value (RFV). However, the starch content significantly increased by 14%, and the ash content significantly decreased by 19%, indicating an improvement in alfalfa quality (Figure 6D,F). Nutritional components in shoots and roots were further analyzed (Figure S1). Compared to the control (Figure S1A,B), NSe significantly increased protein content by 30% in shoots and 15% in roots, while root amino acids significantly increased by 59%. NSe also significantly increased soluble sugars (17%) and flavonoids (13%) in shoots (Figure S1C,E). For the antioxidant system (Figure S1G–J), NSe significantly increased GSH-Px activity by 19% (shoots) and 30% (roots). It significantly elevated GSH content in shoots (27.3%), while significantly reducing GSSG accumulation (5.1% in shoots, 16% in roots), resulting in significantly higher GSH/GSSG ratios (34% in shoots, 54% in roots). Phytohormone analysis (Figure S1K–N) revealed that NSe significantly promoted the accumulation of JA (90%), MeJA (70%), and ABA (59%), but significantly reduced SA (6.5%). Amino acid profiling demonstrated consistent trends between field and pot experiments (Figure S2): eight shoot amino acids (e.g., Gln, Ala) significantly increased by 17–48%, while six root amino acids (e.g., His, Arg) significantly rose by 5.4–47%. Notably, Glu significantly decreased in shoots (17%) and roots (29%) under field conditions.

### 2.7. Metabolite Analysis

**Figure 7:**
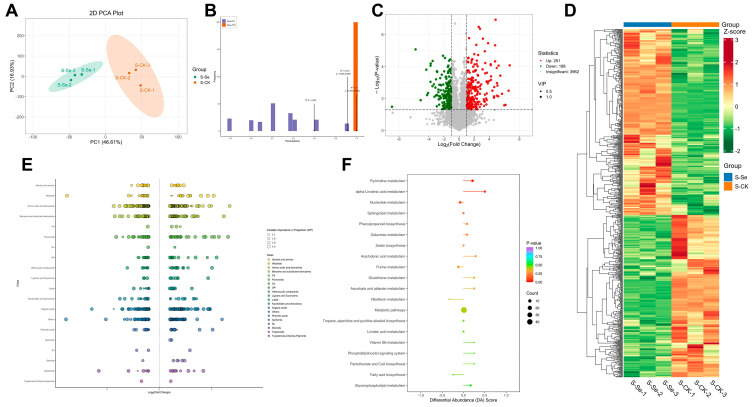
Metabolite profiles in alfalfa shoots under field NSe treatment (**A**) Principal component analysis (PCA), (**B**) OPLS-DA score plot, (**C**) Volcano plot, (**D**) Heatmap, (**E**) Scatter plot of differential metabolites, (**F**) Differential abundance score plot analysis.

**Figure 8:**
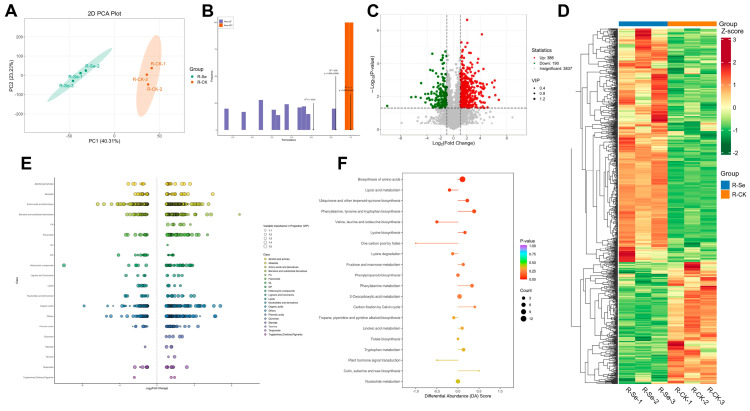
Metabolite profiles in alfalfa roots under field NSe treatment (**A**) Principal component analysis (PCA), (**B**) OPLS-DA score plot, (**C**) Volcano plot, (**D**) Heatmap, (**E**) Scatter plot of differential metabolites, (**F**) Differential abundance score plot analysis.

Three biological replicates per treatment were analyzed for untargeted metabolomic analysis. Untargeted metabolomics was employed to investigate the types and concentrations of metabolites in alfalfa shoots and roots, to elucidate the mechanisms by which NSe modulates alfalfa metabolism. Using UPLC-MS/MS, 4411 and 4413 metabolites were detected in shoots and roots, respectively (Figure 7C and Figure 8C). Principal component analysis (PCA) and orthogonal partial least squares discriminant analysis (OPLS-DA) (Figure 7A,B and Figure 8A,B) demonstrated clear separation between the NSe-treated group and the control group. Compared to the control (Figure 7C and Figure 8C), 449 and 576 differentially abundant metabolites (DAMs) were identified in shoots and roots, respectively, using the criteria of variable importance in projection (VIP) > 1, fold change ≥ 2, or fold change ≤ 0.5. Among these, 251 (shoots) and 386 (roots) DAMs were up-regulated, while 198 (shoots) and 190 (roots) were down-regulated. Cluster heatmaps visualized the screened DAMs (Figure 7D,E and Figure 8D,E), and scatter plots revealed that the differential metabolites were primarily concentrated in amino acids and derivatives, benzene and substituted derivatives, organic acids, and flavonoids. Kyoto Encyclopedia of Genes and Genomes (KEGG) pathway enrichment analysis indicated that NSe treatment primarily influenced shoot growth and material accumulation by modulating pyrimidine metabolism, α-linolenic acid metabolism, nucleotide metabolism, and phenylpropanoid biosynthesis (Figure 7F). Simultaneously, NSe affected root growth and nutrient synthesis by regulating pathways such as amino acid biosynthesis, phenylalanine, tyrosine, tryptophan biosynthesis; valine, leucine, isoleucine biosynthesis; and phenylpropanoid biosynthesis (Figure 8F).

### 2.8. Soil Microbial Analysis

**Figure 9:**
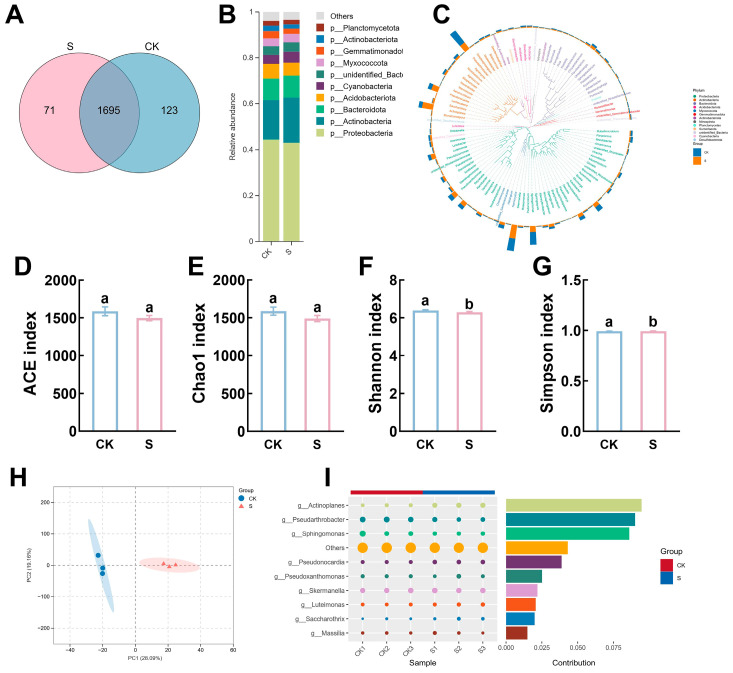
Rhizosphere soil microbiome under field NSe treatment (**A**) Venn diagram of bacterial community distribution, (**B**) Microbial community composition at the phylum level, (**C**) Phylogenetic tree at the genus level, (**D**–**G**) α-Diversity indices (ACE, Chao1, Shannon, Simpson), (**H**) PCA of microbial β-diversity, (**I**) Contribution of differential microbial genera. Data are presented as mean ± SEM. Different letters indicate significant differences at *p* < 0.05.

Three biological replicates per treatment were analyzed for rhizosphere microbial community composition. Foliar application of NSe altered the microbial community in alfalfa rhizosphere soil. As shown in Figure 9A, 123 amplicon sequence variants (ASVs) were unique to the control (CK) group, 71 ASVs were unique to the NSe-treated group, and 1695 ASVs were shared between the CK and NSe groups. The dominant bacterial groups in alfalfa rhizosphere soil included Proteobacteria, Actinobacteria, Bacteroidota, Acidobacteriota, and Cyanobacteria at the phylum level (Figure 9B). After NSe foliar treatment, the relative abundances of Actinobacteria and Cyanobacteria increased, whereas Proteobacteria, Acidobacteriota, and Gemmatimonadota decreased. Compared to the control (Figure 9D–G), NSe treatment did not alter the ACE or Chao1 indices but reduced the Shannon and Simpson indices, indicating a decrease in the α-diversity of the rhizosphere bacterial community. Principal component analysis (PCA) of β-diversity (Figure 9H) revealed significant separation between NSe-treated and control groups, with principal component 1 (PC1) and principal component 2 (PC2) explaining 29% and 19% of the total variance, respectively. At the genus level (Figure 9I), microbes with significant contributions to the differences included *Actinoplanes*, *Pseudarthrobacter*, *Sphingomonas*, and *Pseudonocardia*. Soil physicochemical properties (Figure S3B–D) showed that NSe foliar application significantly decreased soil pH by 0.17 units, significantly increased soil urease activity by 16%, and had no significant effect on soil sucrase activity.

### 2.9. Correlation Analysis

To explore potential associations between metabolites and enzymes under NSe treatment, correlation analyses were conducted (Figures S4 and S5). In pot experiments, soluble protein and amino acids showed significant positive correlations with JA, CAT, GSH-Px, and GSH. Soluble protein exhibited a strong correlation with GSH-Px (|r| ≥ 0.8). Glu displayed moderate positive correlations (0.5 ≤ |r| < 0.8) with His, Arg, Pro, and ABA. Pro showed moderate-to-strong positive correlations (|r| ≥ 0.5) with His, Arg, and ABA. SA demonstrated moderate positive correlations with SOD, POD, GSH-Px, and ASA. JA correlated positively with SOD, CAT, GSH-Px, and GSH, with moderate correlations for all except SOD (weak). Field experiments revealed strong negative correlations between Glu and soluble protein, amino acids, Gln, Ala, His, Pro, ABA, JA, and MeJA. Pro exhibited strong positive correlations with ABA, JA, MeJA, etc. ABA, JA, and MeJA showed significant positive correlations with amino acids including soluble protein, total amino acids, Gln, and Ala.

## 3. Discussion

Recent advances highlight NSe as a promising tool for enhancing plant nutrient fortification and regulating stress resistance. Exogenous Se enhances crop growth through modulation of plant metabolism and microbial communities. However, the mechanisms by which NSe regulates antioxidant-secondary metabolic networks and rhizosphere microenvironment modification to improve alfalfa stress resistance remain insufficiently characterized. Furthermore, stage-specific metabolic responses (seedling vs. field growth) require systematic investigation. Furthermore, molecular pathways underlying selenium-mediated regulation of key forage quality components (e.g., amino acids, proteins) require clarification. Elucidating these multidimensional mechanisms will advance the theoretical frameworks for Se-biofortified forage technology. This understanding will further provide practical strategies to optimize resource utilization and enhance livestock productivity.

### 3.1. Nutrient Metabolism in Alfalfa Under Foliar NSe

As a core protein source for global livestock, alfalfa yield is closely linked to photosynthetic carbon assimilation. This study found that NSe significantly increased chlorophyll and carotenoid levels in seedlings (Figure 1F–I), likely by enhancing the stability of photosystem Ⅱ and improving electron transfer efficiency, thereby improving light energy capture and providing more carbon skeletons and energy for organic synthesis. Field trials further validated the growth-promoting effects of NSe, with plant height and dry weight increasing by 20% and 14%, respectively (Figure 6A,B). Carbohydrates are categorized into structural carbohydrates (SCs, e.g., cellulose, hemicellulose, lignin, and pectin) for cell wall formation and non-structural carbohydrates (NSCs, e.g., free sugars, starch) that play vital roles in plant growth, energy metabolism, source-sink regulation, and stress responses. Higher NSCs also enhance forage intake and digestibility [17]. Starch serves as a primary energy source for livestock, while ADF and NDF reflect indigestible components, including cellulose, hemicellulose, and lignin, which can impair animal digestion when elevated [18]. Foliar application of Se can regulate carbohydrate metabolic pathways, significantly enhancing carbohydrate synthesis and energy metabolism in plants [19]. Moreover, Se treatment markedly increases selenium content in alfalfa silage while reducing NDF and ADF levels, thereby improving its forage quality [20]. Our field trial data (Figure 6, Figure 7 and Figure S1) demonstrate that NSe application enhanced the accumulation of starch (14%) and soluble sugar (16%) in alfalfa shoots. It also stimulated phenylpropanoid biosynthesis, elevating flavonoid levels by 13%. This treatment strategically redirected carbon flux toward these valuable metabolites (starch, sugars, flavonoids) rather than structural fibers, a shift confirmed by stable ADF/NDF values (Figure 6G,H).

Efficient nitrogen utilization is crucial to enhancing alfalfa quality. Crude protein (CP). Crude protein (CP), measured by the Kjeldahl method, reflects total nitrogen content including structural proteins, nucleic acids, and non-protein nitrogen. In contrast, soluble proteins quantified via colorimetric assays represent metabolically active functional proteins, while free amino acids serve as immediate nitrogen pools for protein synthesis. This study revealed that field-applied NSe significantly increased soluble protein content (30% in shoots, 15% in roots) and free amino acid levels (59% in roots) (Figure S1A,B), whereas CP showed no significant change. This discrepancy may reflect a shift in nitrogen allocation patterns, with NSe treatment redistributing nitrogen resources toward metabolically active components. Concurrently, metabolic pathways associated with amino acid synthesis were activated in roots (Figure 8F), with pronounced accumulation of Gln, Arg, Pro, and His observed in both shoots and roots (Figure S2). Similar enhancements in soluble proteins (26%) and amino acids (48%) were detected in alfalfa seedlings (Figure 2A,B and Figure 3). Integrated analysis of metabolomics and amino acid profiling suggests that NSe likely promotes nitrogen assimilation to facilitate the conversion of nitrogen into amino acids, thereby modulating nitrogen allocation and improving nitrogen use efficiency in alfalfa. Li et al. [12] reported that NSe stimulates GS and GOGAT activities, thereby enhancing the GS/GOGAT cycle to drive the accumulation of arginine, glutamate, and proline. Sun et al. [21] corroborated these findings, showing NSe boosts nitrogen assimilation enzymes (GS, nitrate reductase (NR), nitrite reductase (Nir)) and fuels amino acid synthesis via upregulated “starch and sucrose metabolism” and “pentose phosphate pathway”, providing precursors and energy. Enhanced amino acid and protein synthesis also supplies precursors and enzymes for other metabolic pathways. For instance, Gln serves as a precursor for pyrimidine biosynthesis [22], potentially explaining the activated pyrimidine metabolism under NSe (Figure 7F). Although conventional forage parameters (CP, ADF, NDF, RFV) showed no significant alterations, this study detected significant increases in starch, soluble protein, and selected functional amino acids, which may enhance the energy and nutritional value of alfalfa. Concurrently, elevated levels of antioxidants such as flavonoids and GSH might strengthen the antioxidant capacity of the forage. Collectively, these changes may refine nutritional quality for alfalfa, although they have no significant impact on conventional forage parameters.

Amino acids further contribute to stress resistance and nutritional value. Glutamate, a metabolic hub in higher plants, enhances forage palatability and digestion. Li et al. [23] found that supplementing Hu sheep with 3 g/head/day L-Glu improved rumen fermentation, digestion, and immunity. In seedlings, NSe elevated glutamate levels (1–10 mg/L), whereas field-grown alfalfa showed reduced Glu, likely due to environmental differences. Potted experiments were conducted under controlled temperature/humidity without biotic stressors, whereas field trials experienced fluctuating temperatures (12–38 °C), stochastic precipitation, and pest pressures (e.g., thrips). Given the significant negative correlations between Glu and soluble proteins/JA (Figure S5), and considering the role of Glu as a metabolic precursor. We propose that under such environmental stress, NSe may facilitate the conversion of glutamate into stress-related compounds like proteins, thereby enhancing alfalfa stress resilience [24]. Glutamate depletion may reflect its role as a substrate for stress-related compounds (e.g., Pro, GABA) or GSH synthesis [25], as evidenced by activated GSH metabolism and elevated GSH levels in field trials (Figure S1H and Figure 7F). Arginine and glutamine, with high N: C ratios, function as nitrogen storage compounds. Arginine also serves as a precursor to polyamines, such as putrescine and spermidine, which regulate growth and stress responses [26]. Under NSe treatment, alfalfa exhibited significantly increased Arg and Glu levels (Figure 3 and Figure S2). This indicates a dual regulatory mechanism: on the one hand, NSe optimizes nitrogen allocation by prioritizing high N: C ratio amino acids; on the other hand, NSe likely modulates plant growth and stress responses by mediating polyamine biosynthesis. Pro stabilizes proteins, maintains cellular homeostasis, and scavenges radicals, correlating positively with stress resistance [27,28]. Synthesized primarily via the Glu pathway (rate-limited by P5CS under ABA regulation [29]). We observed a significant positive correlation between Pro and ABA (Figures S4 and S5), suggesting that NSe may interact with ABA signaling to modulate Pro accumulation. Met and Lys are critical components for casein synthesis and represent the first-limiting amino acids in protein synthesis for ruminants, such as cattle [30]. Field trials demonstrated that NSe significantly elevated Pro (32%), Gln (31%), and Lys (33%) levels in alfalfa (Figure S2). These findings indicate that NSe enhances alfalfa nutritional value and stress resistance by promoting biomass accumulation through targeted regulation of amino acid composition.

### 3.2. Antioxidant Defense and Stress Hormones in Response to Foliar NSe

Plants require a balanced allocation of resources between growth and defense to ensure normal development. Phytohormones are pivotal in regulating plant growth, stress resistance, and metabolic homeostasis [31]. Field trials revealed that NSe activates the α-linolenic acid metabolism pathway, increasing JA (89%) and MeJA (70%) levels in shoots [32], while reducing SA. In contrast, pot experiments with seedlings under NSe (20 mg/L) showed elevated JA (44%) and SA (94%) but decreased MeJA (45%). MeJA serves as a crucial endogenous signaling molecule in plant insect resistance. It can deter herbivorous insects by inducing the release of plant volatiles. Shuang et al. [33] demonstrated that exogenous MeJA at relatively high concentrations reduced the behavioral preference of thrips toward alfalfa. Since field-grown alfalfa is inevitably exposed to herbivorous insects such as thrips, NSe treatment promotes the accumulation of MeJA, enabling a rapid defensive response upon herbivore attack. JA enhances disease resistance by upregulating defense-related genes and promoting secondary metabolite synthesis [34]. Both JA and MeJA stimulate phenylalanine ammonia-lyase (PAL) and chalcone isomerase (CHI) activities and gene expression, thereby driving polyphenol biosynthesis to combat herbivores and pathogens [35,36]. This study observed upregulated α-linolenic acid and phenylpropanoid metabolism under NSe (Figure 7F), alongside significantly elevated flavonoid content (Figure S1E). These effects may stem from NSe-induced JA/MeJA accumulation, which further promotes phenylpropanoid compound synthesis, thereby strengthening the stress resistance of alfalfa. Antioxidant systems serve as critical defenses against environmental stress. Antioxidant systems serve as critical defenses against environmental stress [37]. SA alleviates zinc toxicity by inducing POD/SOD activities and enhancing glutathione metabolism [38]. We also observed a significant positive correlation between SA and the activities of SOD, POD, and GSH-Px in our pot experiment (Figure S4). In this study, NSe increased SOD, POD, and CAT activities (Figure 5C–E), synergistically mitigating ROS-induced cellular damage—consistent with its roles in wheat [39] and pepper [40]. GSH-Px catalyzes the conversion of reduced GSH to GSSG while reducing harmful peroxides to non-toxic hydroxyl compounds and regenerates GSH via glutathione reductase (GR). Li et al. [12] demonstrated that NSe enhances the ASA-GSH cycle by boosting enzyme activities (APX, DHAR, and GR) and antioxidant levels, aiding detoxification. Field trials confirmed that NSe elevates GSH-Px activity, GSH content, and GSH/GSSG ratios (Figure S1), optimizing glutathione metabolism and ascorbate metabolism to improve antioxidant buffering capacity, aligning with the findings of He et al. [41], where Se enhanced GR activity to maintain GSH/GSSG homeostasis. The ASA-GSH cycle is further modulated by JA, which mitigates oxidative damage under cold stress by regulating ASA-GSH functionality [42]. We also detected a significant positive correlation between JA and both GSH and GSH-Px (Figure S4), suggesting that NSe may influence ASA-GSH-related antioxidant defense partly through the regulation of JA biosynthesis. Collectively, NSe may enhance stress resistance potential through coordinated phytohormone signaling and antioxidant system regulation. Critically, the observed enhancement in stress tolerance must be interpreted as an indirect inference from phytohormone and antioxidant biomarker shifts, not as direct evidence of resistance, since no direct stress bioassays (e.g., controlled drought or pathogen challenge) were conducted.

### 3.3. Rhizosphere Microenvironment Alterations Under Foliar NSe

Rhizosphere microbial communities mediate plant–soil interactions. DNA sequencing revealed Proteobacteria and Actinobacteria as the dominant phyla in alfalfa rhizosphere soil. Notably, NSe application altered microbial community structure: Actinobacteria relative abundance increased while Proteobacteria decreased (Figure 9), concomitant with reduced soil pH and elevated urease activity. As root exudates and Se root translocation were not directly measured, we propose three non-exclusive pathways: (1) Drift during drone spraying introduced NSe directly into soil (rhizosphere Se content increased by 27% in NSe-treated soil, Figure 6C); (2) Foliar-absorbed Se translocated to roots via phloem, as reported by Wang et al. [43]; (3) NSe-modulated hormone signaling and carbon-nitrogen metabolism altered root exudate composition. At the genus level (Figure S3A), NSe foliar application increased abundances of *Actinoplanes* and *Pseudonocardia* (both Actinobacteria). *Pseudonocardia* is known for producing antibiotics and facilitating the degradation of environmental pollutants [44]. Rhizosphere microbial communities interact with soil physicochemical properties. Alfalfa thrives in slightly acidic soils [45], Alfalfa thrives in slightly acidic soils, while the field soil pH was 8.7. The NSe-induced pH reduction may thus better suit alfalfa growth. This pH decline may be linked to acid-producing metabolism of Actinobacteria and modified root exudation patterns. Legume-specific nitrogen fixation processes also intensify rhizosphere acidification, with studies linking a decline in soil pH to elevated nitrogen content [46], suggesting that NSe-induced pH changes may reflect enhanced nitrogen fixation. We also detected higher soil urease activity under NSe, consistent with Li et al. [47] and Zhou et al. [48] Urease activity correlates with organic nitrogen mineralization, and its elevation indicates improved soil nitrogen availability. Especially under low nitrogen soil conditions (746 mg/kg). NSe may promote nitrogen storage via activated root amino acid synthesis pathways, ensuring nutritional supply. Although reduced α-diversity is generally considered detrimental to soil resilience, its co-occurrence with functional taxa (Actinobacteria) enrichment and enhanced nitrogen-transforming enzymes in this study may indicate community-level functional specialization toward nitrogen cycling. Crucially, as root exudates and Se root translocation were unmeasured, the causal relationship between foliar NSe and observed microbial shifts requires further validation through Se-tracing studies and root exudate profiling.

Despite the multidimensional benefits of NSe, potential environmental risks from long-term application warrant careful consideration. Continuous use may lead to selenium accumulation in soil-water systems, posing ecological risks to sensitive organisms (e.g., earthworms, waterfowl, fish) [49]. Additionally, plant-to-feed-to-food chain transfer could elevate dietary selenium exposure in humans, potentially causing excessive accumulation. In this study, post-treatment rhizosphere soil selenium content was 0.053 mg/kg, below the deficiency threshold (<0.125 mg/kg) [50]. However, given China’s significant spatial heterogeneity in Se distribution and ecological effects, site-specific data cannot be generalized. Future research should monitor selenium speciation and bioavailability dynamics under prolonged application and develop regionally tailored ecological and health risk management strategies.

## 4. Materials and Methods

### 4.1. Plant Cultivation

Pot experiments were conducted in a greenhouse at the College of Resources and Environmental Sciences, China Agricultural University. Soil for pot experiments was collected from the surface layer (0–20 cm) of experimental fields in Ordos City, Inner Mongolia Autonomous Region, China (40°24′ N, 109°53′ E). After air-drying and sieving (2 mm), the soil was identified as Castanozems with the following properties: pH 8.7, organic matter 12.5 g/kg, total nitrogen 746.1 mg/kg, available phosphorus 23.3 mg/kg, and available potassium 93.3 mg/kg. Alfalfa seeds were rinsed three times with distilled water, surface-sterilized with 1% NaClO for 30 min, and then washed five times with deionized water. Germination was carried out on two layers of moist filter paper in 9 cm Petri dishes at 25 °C in darkness for 2 days. Seedlings were transplanted into plastic pots (20 cm diameter × 18 cm depth) filled with 2 kg soil, with seven plants per pot. Greenhouse conditions were controlled at: temperature (25/20 °C day/night), relative humidity (60–80%), light/dark cycle (14/10 h), and light intensity (250–300 μmol·m^−2^·s^−1^). Distilled water (100 mL) was replenished every day to maintain soil moisture. The first foliar NSe was applied at the third actual leaf stage, followed by treatments every 6 days. The synthesis methodology of NSe nanoparticles and their characterization data (Figure S6) are provided in the Supplementary Materials. Dynamic light scattering analysis revealed that the NSe particles exhibited an average diameter ranging from 50 to 78 nm [51]. Sampling was conducted 17 days after the final treatment. Aboveground tissues were flash-frozen in liquid nitrogen for physiological and biochemical analyses.

Field trials were conducted from July to September 2024 in Dalad Banner, Ordos City, Inner Mongolia Autonomous Region (40°24′ N, 109°53′ E), using the alfalfa cultivar WL712 (fall dormancy rating 10). The experimental field soil had similar physicochemical properties to those of the pot experiment soil. Detailed protocols for alfalfa growth stages, treatment schedules, and meteorological data during the field trial are provided in Supporting Materials. NSe stock solution (2500 mg/L) was diluted 60 times with distilled water (final concentration: 41.7 mg/L) and applied via foliar spray using an agricultural drone (90 L/ha). Control plots received an equivalent amount of deionized water. Three consecutive treatments were administered. Sampling occurred 8 days after the final treatment (early flowering stage), with nine sampling points (3 m × 3 m each) arranged in a 3 × 3 grid per treatment. Samples from the nine points were pooled to form three biological replicates. Samples were chopped, flash-frozen using dry ice, and transported to China Agricultural University for analysis. Rhizosphere soil was collected by shaking roots to retain soil within 5 mm of the root surface. Three sampling points per treatment were pooled, sieved (2 mm), and stored at −80 °C for microbial analysis or air-dried for physicochemical property determination. Detailed information on materials, standards, and reagents is presented in the Supplementary Materials.

### 4.2. Alfalfa Feeding Value Analysis

Harvested aboveground tissues were chopped, heat-inactivated at 110 °C for 10 min, and dried at 65 °C to a constant weight. Dried samples were cooled in a desiccator, ground, and sieved (0.5 mm) for analysis. Starch, CP, and ash content were determined using acid hydrolysis-DNS, Kjeldahl, and acid digestion methods. ADF and NDF were measured via gravimetric washing.

### 4.3. Total Selenium Content Analysis

Total Se in dried alfalfa tissues and air-dried rhizosphere soil was quantified according to the Chinese National Standard (GB 5009.268-2016) [52].

### 4.4. Photosynthetic Pigment Analysis

Photosynthetic pigments (chlorophyll and carotenoids) were extracted using ethanol/acetone and quantified via UV spectrophotometry following Yu et al. [53].

### 4.5. Enzyme Activity Analysis

Soluble protein, free amino acids, soluble sugars, total flavonoids (TF), total phenolics (TP), total antioxidant capacity (DPPH), malondialdehyde (MDA), glutathione (GSH), oxidized glutathione (GSSG), ascorbic acid (ASA), and enzyme activities (Superoxide dismutase (SOD), Peroxidase (POD), Catalase (CAT), soil urease (S-UE), and soil sucrase (S-SC)) were analyzed using commercial kits (Suzhou Comin Biotechnology Co., Ltd., Suzhou, China). GSH-Px activities were analyzed using commercial kits (Nanjing Jiancheng Bioengineering Institute, Nanjing, China).

### 4.6. Amino Acid Analysis

Water-soluble amino acids were measured in accordance with GB/T 30987-2020 [54]. Frozen alfalfa powder (1:125 *w*/*v*) was extracted in boiling deionized water (95 °C, 10 min), filtered (0.45 μm), and analyzed via Agilent 6465 Triple Quadrupole UPLC-MS/MS (Ultivo, Santa Clara, CA, USA) equipped with an Eclipse Plus C18 column (2.1 mm × 50 mm, 1.8 μm). Instrument parameters are detailed in Tables S1 and S2.

### 4.7. Phytohormone Analysis

Phytohormone levels in alfalfa were measured according to the method described by Zhou et al. [48]. Briefly, 100 mg of frozen alfalfa powder was extracted with 1 mL of an 80% aqueous methanol solution containing 1% formic acid. The mixture was ultrasonicated in an ice bath for 10 min and then centrifuged at 4 °C and 12,000 rpm for 5 min. The extraction was repeated once, and the supernatants were combined. The pooled supernatant was purified with 100 mg PSA adsorbent and filtered through a 0.22 μm organic phase filter membrane. The filtrate was analyzed using an Agilent 6465 Triple Quadrupole UPLC-MS/MS system (Ultivo, Santa Clara, CA, USA) equipped with an Agilent Eclipse Plus C18 column (2.1 mm × 50 mm, 1.8 μm; Agilent, Santa Clara, CA, USA). Detailed instrument parameters are provided in Tables S3 and S4.

### 4.8. Metabolomics Analysis

Alfalfa samples were freeze-dried using a Scientz-100F freeze dryer and ground into powder with an MM 400 grinding mill (Retsch, Haan, Germany). A total of 50 mg of powder was weighed using an MS105DM electronic balance and mixed with 1200 μL of pre-cooled (−20 °C) 70% methanol aqueous solution containing internal standards. (For internal standard preparation: 1 mg of reference standard was dissolved in 1 mL of 70% methanol to prepare a 1000 μg/mL stock solution, which was further diluted with 70% methanol to obtain a 250 μg/mL working solution.) The mixture was vortexed six times (30 s each at 30 min intervals) and centrifuged at 12,000 rpm for 3 min. The supernatant was filtered through a 0.22 μm microporous membrane and analyzed using a Shimadzu LC-30A ultra-performance liquid chromatography system (Kyoto, Japan) coupled with a SCIEX TripleTOF 6600+ mass spectrometer (Foster City, CA, USA) and a Waters ACQUITY UPLC HSS T3 column (1.8 μm, 2.1 mm × 100 mm) (Milford, MA, USA). Instrument parameters are detailed in Tables S5 and S6, while the Non-targeted Metabolomics Raw Data are available in Supplementary Table S7.

### 4.9. Microbiome Analysis

Rhizosphere soil microbial communities were analyzed via 16S rDNA high-throughput sequencing. DNA extraction, PCR amplification, product purification, library preparation and quality control, sequencing, and data analysis along with the corresponding analytical platforms, are provided in the Supplementary Materials. The V4-V5 regions of bacterial 16S rDNA genes were amplified using primers 515F (5′-GTGCCAGCMGCCGCGGTAA-3′) and 907R (5′-CCGTCAATTCCTTTGAGTTT-3′). The top 10 abundant microbial taxa are presented in Supplementary Table S8.

### 4.10. Statistical Analysis

Data were analyzed using Microsoft Excel 2019 and IBM SPSS Statistics 26. Data were first tested for normality (Shapiro–Wilk test) and homogeneity of variances (Levene’s test). When parametric assumptions were met, one-way ANOVA followed by Duncan’s test was performed for significance analysis (*p* < 0.05), with differences indicated by letters (a, b, c, etc.). Figures were generated and annotated using PowerPoint 2019, GraphPad Prism 9, OriginPro 2024, and the Metware Cloud platform (https://cloud.metware.cn (accessed on 27 February 2025)).

## 5. Conclusions

This study employed pot and field experiments to elucidate the multidimensional regulatory mechanisms of foliar-applied NSe on alfalfa growth and metabolism. Results demonstrated that in pot trials, NSe at 1–20 mg/L enhanced alfalfa growth and stress tolerance by modulating nitrogen metabolism, activating antioxidant systems, and regulating hormone signaling. Field experiments validated the cross-environment stability of NSe-mediated regulatory mechanisms, showing consistent outcomes with pot trials. Metabolomic analysis revealed that NSe differentially regulated shoot-root metabolic networks. Shoots exhibited activated pyrimidine metabolism, α-linolenic acid metabolism, and phenylpropanoid metabolism. Whereas roots were enriched in amino acid biosynthesis pathways (phenylalanine, tyrosine, tryptophan biosynthesis and lysine biosynthesis), achieving synergistic shoot-root co-enhancement. NSe enhanced alfalfa nutritional quality through improved nitrogen use efficiency. This significantly elevated soluble protein and functional amino acid content (notably Glu, Arg, His, Pro). Concurrently, NSe promoted starch and soluble sugar synthesis via carbon metabolic reprogramming. However, it did not improve conventional forage quality indicators. Concurrently, NSe foliar application was associated with modulations in hormone signaling (ABA, JA, MeJA, and SA) and enhanced antioxidant capacity (SOD, POD, CAT, GSH-Px, and GSH), potentially improving alfalfa physiological adaptation. Additionally, rhizosphere microenvironment alterations included Actinobacteria enrichment and elevated soil urease activity. Critically, the claims of enhanced stress resistance are inferred indirectly from antioxidant and hormonal biomarkers, as no direct stress challenges were applied in this experimental design. While this study focused on a single alfalfa variety and exogenous substance, future research should expand to multi-variety and multi-substance application strategies, elucidate exogenous agent-mediated metabolic reprogramming and epigenetic mechanisms, and develop exogenous formulations for combined stress environments, providing theoretical and technical foundations for eco-friendly forage enhancement.

## Data Availability

The data presented in this study are available on request from the corresponding author.

## Supplementary Materials

The following supporting information can be downloaded at: https://www.mdpi.com/article/10.3390/ijms26189013/s1.
