# Supplementary material for: Foliar Nano-Selenium Modulates Metabolic and Antioxidant Responses in Alfalfa (Medicago sativa L.): Integration of Pot and Field Evidence
Source: Int J Mol Sci. 2025 Sep 16;26(18):9013. doi: 10.3390/ijms26189013 (PMC12469622; doi:10.3390/ijms26189013)
Supplement: Supplementary file 1 [file ijms-26-09013-s001.zip › Supplementary Materials for Figures, Tables, and Methods.pdf]

Foliar Nano-Selenium Modulates Metabolic and Antioxidant Responses in Alfalfa (*Medicago sativa* L.): Integration of Pot and Field Evidence

Haiyan Cheng [a], Huan Yu [a], Qinyong Dong [a], Chunran Zhou [a], Tingjie Huang [a], Xun Fang [a] and Canping Pan [a, *].

[a] Innovation Center of Pesticide Research, Department of Applied Chemistry, College of Science, China Agricultural University, Beijing 100193, China Yuanmingyuan West Road 2, Beijing 100193, PR China.

***Corresponding author:**

Canping Pan: canpingp@cau.edu.cn

Authors:

Haiyan Cheng: haiyancheng@cau.edu.cn

Huan Yu: yhteawork@163.com

Qinyong Dong: dontinue@126.com

Chunran Zhou: chunranzhou@cau.edu.cn

Tingjie Huang: takeyourtimehtj@163.com

Xun Fang: fangxun@cau.edu.cn

Total number of pages: 17

Total number of figures: 6

Total number of tables: 6

1. SUPPORTING MATERIALS

1.1. Materials, Standards, and Reagents.

Analytical ethanol was purchased from Bei Jing Tong Guang Fine Chemicals Company (Beijing, China). Formic acid, acetonitrile, methanol, and acetone (chromatographic pure) were acquired from Fisher Chemicals (Fair Lawn, NJ, USA). Octadecylsilane (C18) and primary secondary amine (PSA) were obtained from HAMAG Instrument Technology Co., Ltd. (Ningbo, China). NaOH (analytical grade) was provided by Sinopharm Chemical Reagent Co., Ltd. (Beijing, China). ALA, ARG, ASN, ASP, CYS, GLU, GLN, GLY, HIS, ILE, LEU, LYS, MET, PHE, PRO, SER, THR, TRP, TYR, VAL (97–99%, technical grade) were obtained from Macklin Inc. (Shanghai, China). Deionized water was supplied by the Wahaha Company (Hangzhou, China).

1.2 Synthesis of NSe

A 1% chitosan solution was prepared as the precursor. Subsequently, a 20 mM selenium dioxide (selenite) solution was gradually added to 20 mL of the precursor under sustained mechanical agitation (500 rpm, 25 ℃), yielding a nanoscale dispersed colloidal suspension. Following this, 4 mL of 1% ascorbic acid was introduced dropwise into the mixture. The reaction agitated for 3 hours turned transparent red, indicating successful nano-selenium formation.

1.3 Size and Morphology Characterization of NSe

The hydrodynamic diameter and size distribution of the NSe were determined via dynamic light scattering utilizing a Zetasizer Nano ZS90 (Malvern Instruments Corporation, Manchester, U.K.). To confirm particle size and visualize morphology, transmission electron microscopy (TEM) was performed using a JEOL-2100F instrument operating at 200 kV. These analyses yielded an average particle size for the NSe ranging from 50 to 78 nm.[1]

1.4 Alfalfa growth and treatment application schedules

The experimental site experiences a temperate continental climate with a mean annual temperature of 6.1–7.1 ℃, mean annual sunshine duration of approximately 3,000 hours, and a frost-free period of 135–150 days. Located on the Inner Mongolia

Plateau (altitude: 980 m), the region recorded maximum and minimum temperatures of 38 ℃ and 12 ℃, respectively, with total precipitation of 96.5 mm during June–August 2024. Field management protocols included: irrigation every 3–4 days via center-pivot system (600 m^3 per hectare per application); no pesticide application for pest/disease control; and monthly applications of Yuanfengtai organic liquid fertilizer (150 kg per hectare) split into four equal doses. The soil was identified as Castanozems with the following properties: pH 8.7, organic matter 12.5 g/kg, total nitrogen 746.1 mg/kg, available phosphorus 23.3 mg/kg, and available potassium 93.3 mg/kg.

The alfalfa, planted in June 2022 and harvested four times annually, had undergone two pre-experimental harvests, with the most recent on June 3, 2024. The initial NSe application was conducted on 13 July 2024, followed by subsequent treatments on 22 July and 16 August, with sampling completed on 24 August 2024.

1.5 Analysis of Soil Microbiome

Total genome DNA from samples was extracted using CTAB method. DNA concentration and purity was monitored on 1% agarose gels. The V4-V5 region of the bacterial 16S rRNA gene was amplified using PCR(Primers 515F，Sequences (5’-3’) GTGCCAGCMGCCGCGGTAA; Primers 907R ， Sequences (5’-3’) CCGTCAATTCCTTTGAGTTT); All PCR reactions were carried out with 15 μL of Phusion® High -Fidelity PCR Master Mix (New England Biolabs); 2 μM of forward and reverse primers, and about 10 ng template DNA. Thermal cycling consisted of initial denaturation at 98 ℃ for 1 min, followed by 30 cycles of denaturation at 98 °C for 10 s, annealing at 50 ℃ for 30 s, and elongation at 72 ℃ for 30 s. Finally, 72 ℃ for 5 min.

Sequencing libraries were generated using TruSeq® DNA PCR-Free Sample Preparation Kit (Illumina,USA) following manufacturer’s recommendations and index codes were added. The library quality was assessed on the Qubit@ 2.0 Fluorometer (Thermo Scientific) and Agilent Bioanalyzer 2100 system. At last, the library was sequenced on an Illumina NovaSeq platform and 250 bp paired-end reads were generated. Quality filtering on the raw tags were performed under specific filtering

conditions to obtain the high quality clean tags according to the fastp (https://github.com/OpenGene/fastp (accessed on 8 December 2024)). Paired-end reads were merged using FLASH (http://ccb.jhu.edu/software/FLASH/ (accessed on 8 December 2024)). The tags were compared with the reference database (Silva database (16S/18S) https://www.arb-silva. de/ (accessed on 8 December 2024); Unite Database(ITS), https://unite.ut.ee/ (accessed on 8 December 2024)) using UCHIME Algorithm (http://www.drive5.com/usearch/ manual/uchime_algo.html (accessed on 8 December 2024)) to detect chimera sequences, and then the chimera sequences were removed. Then the Effective Tags finally obtained. Amplicon sequence variant (ASV) were analysised by Deblur, which uses error profiles to obtain putative error-free sequences from Illumina sequencing platform. For each representative sequence, the Silva Database (http://www.arb-silva.de/ (accessed on 8 December 2024)) was used based on Mothur algorithm to annotate taxonomic information.

Reference

1. Li, D.; An, Q.; Wu, Y.; Li, J.-Q.; Pan, C. Foliar Application of Selenium Nanoparticles on Celery Stimulates Several Nutrient Component Levels by Regulating the α-Linolenic Acid Pathway. *ACS Sustainable Chemistry & Engineering* **2020**, *8*, 10502-10510. DOI: 10.1021/acssuschemeng.0c02819.

2. SUPPORTING FIGURES AND TABLES

Figure S1. Effects of nano-selenium (NSe) treatment on key quality components, antioxidant parameters and phytohormones in shoots and roots of field-grown alfalfa

Figure S2. Effects of NSe treatment on Free amino acid in shoots and roots of field-grown alfalfa

Figure S3. Effects of NSe treatment on Rhizosphere soil properties of field-grown alfalfa

Figure S4. Pearson correlation analysis between metabolites and enzyme activities in the pot experiment

Figure S5. Pearson correlation analysis between metabolites and enzyme activities in the field experiment

Figure S6. The characterization diagrams of NSe

Table S1. Characteristics of the 20 amino acids determined by HPLC

Table S2. Characteristics of the 20 amino acids determined by HPLC-MS/MS

Table S3. Characteristics of phytohormones determined by HPLC

Table S4. Characteristics of phytohormones determined by UPLC-MS/MS

Table S5. Characteristics of the metabolomics determined by HPLC

Table S6. Mass spectrometry conditions for the AB TripleTOF 6600 system in metabolomics analysis

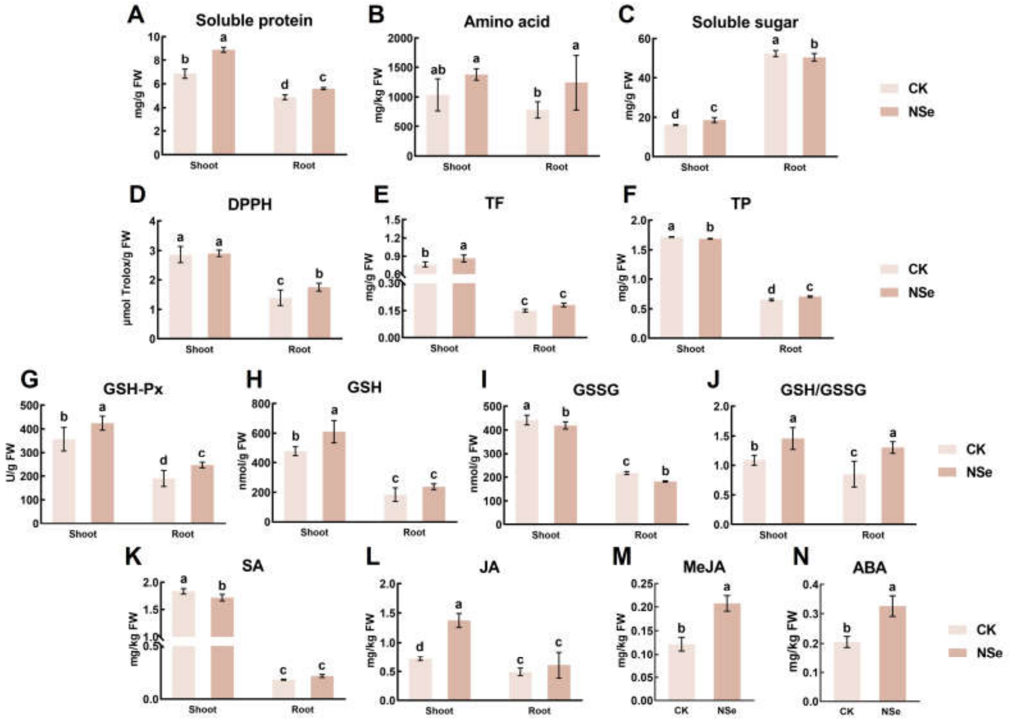

Figure S1. Shoots and roots of field-grown alfalfa under NSe treatment (A)Soluble protein content; (B)Amino acid content; (C)Soluble sugar content; (D) DPPH radical scavenging activity; (E)Tatal Flavonoids (TF) content; (F) Total Phenols (TP) content; (G) Glutathione peroxidase (GSH-Px) activity; (H) Glutathione (GSH) content; (I) oxidized glutathione (GSSG) content; (J)GSH/GSSG; (K) Salicylic Acid (SA) content; (L) Jasmonic Acid (JA) content. And shoots of field-grown alfalfa under NSe treatment: (M) Methyl Jasmonate (MeJA) content; (N) Abscisic Acid (ABA) content. Data are presented as mean±SEM. Different lowercase letters indicate significant differences at $p < 0.05$.

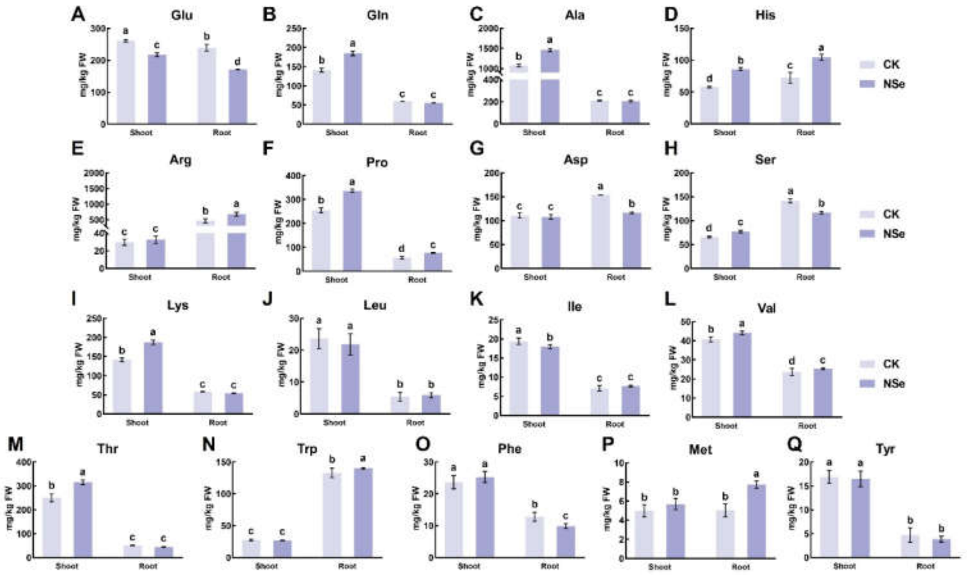

Figure S2. Shoots and roots of field-grown alfalfa under NSe treatment (A) Glutamic acid (Glu) content; (B) Glutamine (Gln); (C) Alanine (Ala) content; (D) Histidine (His) content; (E) Arginine (Arg) content; (F) Proline (Pro) content; (G) Aspartic acid (Asp) content; (H) Serine (Ser) content; (I) Lysine (Lys) content; (J) Leucine (Leu) content; (K) Isoleucine (Ile) content; (L) Valine (Val) content; (M) Threonine (Thr) content; (N) Tryptophan (Trp) content; (O) Phenylalanine (Phe) content; (P) Methionine (Met) content; (Q) Tyrosine (Tyr) content. Data are presented as mean ± SEM. Different lowercase letters indicate significant differences at $p < 0.05$.

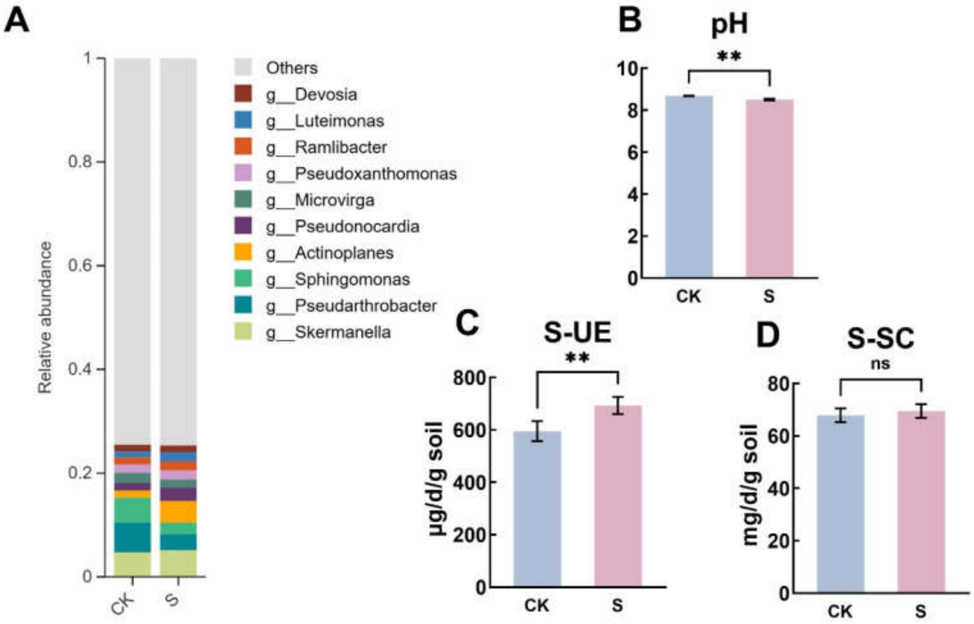

Figure S3. Rhizosphere soil of field-grown alfalfa under NSe treatment: (A) Genus-level microbial community composition; (B) pH; (C) Soil urease activity; (D) Soil sucrase activity. Data are presented as mean ± SEM. Asterisks indicate statistical significance: ** $p < 0.01$, ns = not significant.

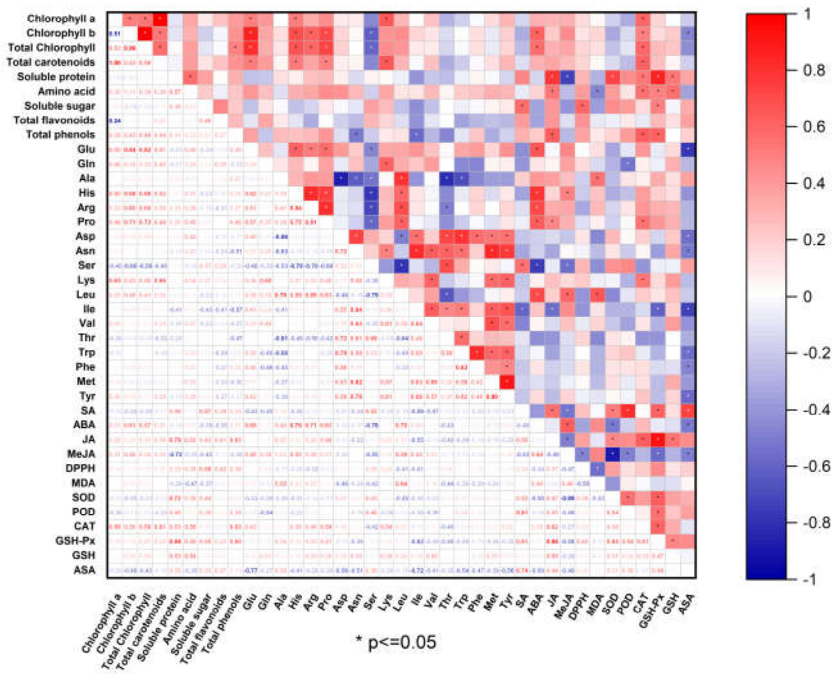

Figure S4. Pearson correlation analysis between metabolites and enzyme activities in the pot experiment is presented as a heatmap. Red indicates positive correlations, blue indicates negative correlations, with color intensity proportional to the correlation coefficient magnitude. Numerical values denote correlation coefficients (where larger |r| values indicate stronger correlations), and asterisks (*) signify statistical significance at $p < 0.05$.

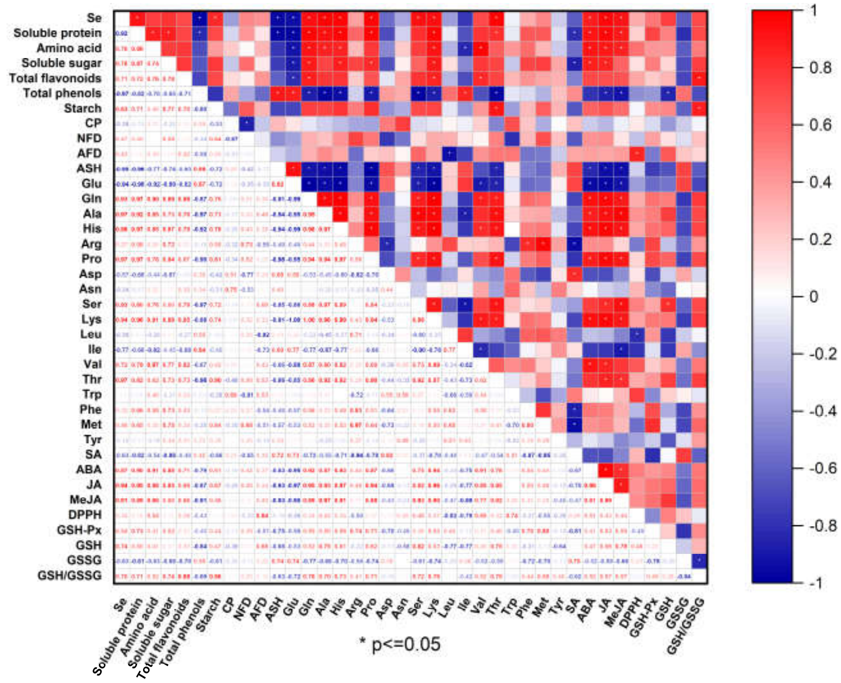

Figure S5. Pearson correlation analysis between metabolites and enzyme activities in the field experiment is presented as a heatmap. Red indicates positive correlations, blue indicates negative correlations, with color intensity proportional to the correlation coefficient magnitude. Numerical values denote correlation coefficients (where larger |r| values indicate stronger correlations), and asterisks (*) signify statistical significance at $p < 0.05$.

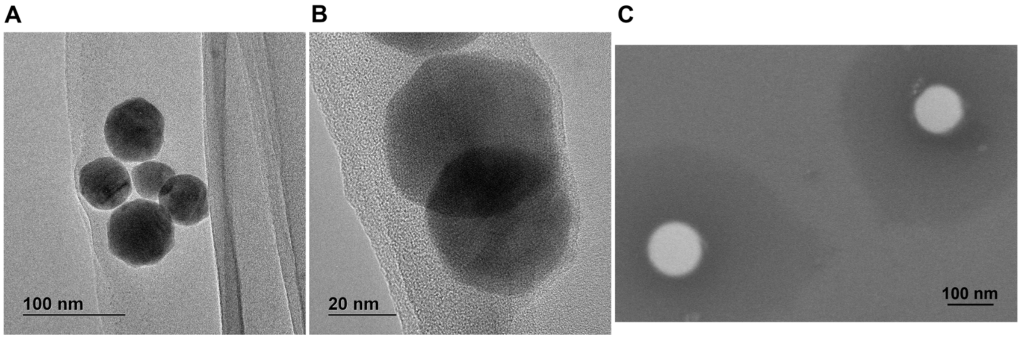

Figure S6. (A) Typical TEM image; (B) HRTEM of an individual NSe; (C) Typical SEM image of NSe.

Table S1. Characteristics of the 20 amino acids determined by HPLC

**Time (min)**	**Flow (mL/min)**	**%A**	**%B**
0.00	0.30	95.00	5.00
1.00	0.30	95.00	5.00
3.00	0.30	76.00	24.00
4.00	0.30	76.00	24.00
6.00	0.30	5.00	95.00
7.00	0.30	5.00	95.00
7.10	0.30	95.00	5.00
8.20	0.30	95.00	5.00

Mobile phases: A: 0.1% formic acid aqueous solution; B: acetonitrile.

Table S2. Characteristics of the 20 amino acids determined by HPLC-MS/MS

Compound	parent ion	qualitative	quantitative	Retention	Fragmentor(V)	CE(V)	Polarity
**Ala**	90	43.9	43.9	1.042	50	5	Positive
**Arg**	175.1	116.1	70.1	0.956	95	5/17	Positive
**Asn**	133.1	74	87.1	2.097	70	1/7	Positive
**Asp**	134	88	74	1.039	65	1/5	Positive
**Cys**	241.1	152	73.9	1.011	90	2/20	Positive
**Gln**	146.8	129.5	83.6	1.038	45	5/15	Positive
**Glu**	148.1	129.8	83.9	1.053	60	5/12	Positive
**Gly**	76	30.1	48.1	1.026	55	1/1	Positive
**His**	156.1	83.1	110.1	0.949	80	5/20	Positive
**Ile**	132	69	86	2.097	35	5/18	Positive
**Leu**	147	44.1	86	2.097	50	5/25	Positive
**Lys**	147	130.1	84	1.038	60	5/16	Positive
**Met**	150	132.9	103.9	1.610	60	5/5	Positive
**Phe**	166	102.8	119.9	3.407	50	10/30	Positive
**Pro**	116.1	43.1	69.9	1.136	65	15/30	Positive
**Ser**	106.1	41.9	59.9	1.026	55	8/20	Positive
**Thr**	119.8	73.8	55.9	1.048	50	5/15	Positive
**Trp**	205	146/118	187.9	4.074	65	8/15/30	Positive
**Tyr**	182	164.9	136.2	1.895	60	5/10	Positive
**Val**	117.9	54.9	71.8	1.311	45	8/20	Positive

Table S3. Characteristics of phytohormones determined by HPLC

Time (min)	Flow (mL/min)	%A	%B
0.00	0.25	95.00	5.00
3.00	0.25	0.00	100.00
4.00	0.25	0.00	100.00
4.01	0.25	95.00	5.00
5.50	0.25	95.00	5.00

Mobile phases: A: 0.1% formic acid aqueous solution; B: acetonitrile.

Table S4. Characteristics of phytohormones determined by UPLC-MS/MS

**Compound**	**parent ion**	**qualitative**	**quantitative**	**Retention**	**Fragmentor(V)**	**CE(V)**	**Polarity**
**ABA**	263.2	219.2	153.1	4.094	90	10/10	Negative
**JA**	209.2	59.1	59.1	4.494	75	5	Negative
**SA**	137.1	93.1	93.1	4.623	55	15	Negative
**MeJA**	225.2	133.1	151.1	5.350	60	12/12	Positive

ABA and MeJA were not detected in alfalfa roots because their concentrations were below the method detection limits.

Table S5. Characteristics of the metabolomics determined by HPLC

**Time (min)**	**Flow (mL/min)**	**%A**	**%B**
0.00	0.40	95.00	5.00
5.00	0.40	35.00	65.00
6.00	0.40	1.00	99.00
7.50	0.40	1.00	99.00
7.60	0.40	95.00	5.00
10.00	0.40	95.00	5.00

Table S6. Mass spectrometry conditions for the AB TripleTOF 6600 system in metabolomics analysis

**Parameters**	**ESI+**	**ESI-**
Duration (min)	10	10
IonSpray Voltage (V)	5000	-4000
Temperature (°C)	550	550
Ion Source Gas1 (psi)	50	50
Ion Source Gas2 (psi)	60	60
Curtain Gas (psi)	35	35
Declustering Potential (V)	60	-60
MS1 Collision Energy (V)	10	-10
MS2 Collision Energy (V)	30	-30
Collision Energy Spread (V)	15	15
MS1 TOF Masses (Da)	50~1250	50~1250
MS2 TOF Masses (Da)	25~1250	25~1250
